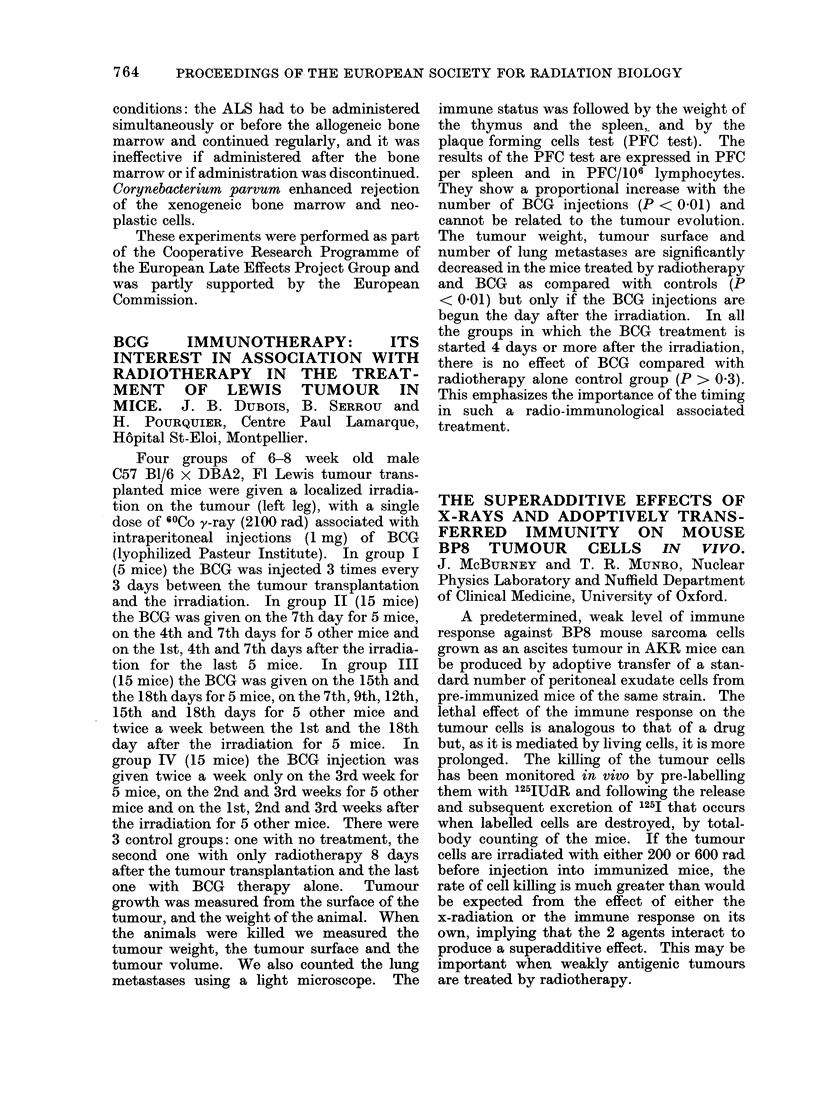# Proceedings: The superadditive effects of x-rays and adoptively transferred immunity on mouse BP8 tumour cells in vivo.

**DOI:** 10.1038/bjc.1975.333

**Published:** 1975-12

**Authors:** J. McBurney, T. R. Munro


**THE SUPERADDITIVE EFFECTS OF X-RAYS AND ADOPTIVELY TRANSFERRED IMMUNITY ON MOUSE BP8 TUMOUR CELLS *IN VIVO*.** J. McBurney and T. R. Munro, Nuclear Physics Laboratory and Nuffield Department of Clinical Medicine, University of Oxford.

A predetermined, weak level of immune response against BP8 mouse sarcoma cells grown as an ascites tumour in AKR mice can be produced by adoptive transfer of a standard number of peritoneal exudate cells from pre-immunized mice of the same strain. The lethal effect of the immune response on the tumour cells is analogous to that of a drug but, as it is mediated by living cells, it is more prolonged. The killing of the tumour cells has been monitored *in vivo* by pre-labelling them with $^{125}$IUdR and following the release and subsequent excretion of $^{125}$I that occurs when labelled cells are destroyed, by total-body counting of the mice. If the tumour cells are irradiated with either 200 or 600 rad before injection into immunized mice, the rate of cell killing is much greater than would be expected from the effect of either the x-radiation or the immune response on its own, implying that the 2 agents interact to produce a superadditive effect. This may be important when weakly antigenic tumours are treated by radiotherapy.